# Boosting the signal-to-noise of low-field MRI with deep learning image reconstruction

**DOI:** 10.1038/s41598-021-87482-7

**Published:** 2021-04-15

**Authors:** N. Koonjoo, B. Zhu, G. Cody Bagnall, D. Bhutto, M. S. Rosen

**Affiliations:** 1 Athinoula A. Martinos Center for Biomedical Imaging, Department of Radiology, Massachusetts General Hospital, Charlestown, MA 02129 USA; 2 Harvard Medical School, Boston, MA 02115 USA; 3 Department of Biological and Agricultural Engineering, Texas A&M University, College Station, TX 77843 USA; 4 Department of Biomedical Engineering, Boston University, Boston, MA 02215 USA; 5 Department of Physics, Harvard University, Cambridge, MA 02138 USA

**Keywords:** Medical imaging, Magnetic resonance imaging, Magnetic resonance imaging

## Abstract

Recent years have seen a resurgence of interest in inexpensive low magnetic field (< 0.3 T) MRI systems mainly due to advances in magnet, coil and gradient set designs. Most of these advances have focused on improving hardware and signal acquisition strategies, and far less on the use of advanced image reconstruction methods to improve attainable image quality at low field. We describe here the use of our end-to-end deep neural network approach (AUTOMAP) to improve the image quality of highly noise-corrupted low-field MRI data. We compare the performance of this approach to two additional state-of-the-art denoising pipelines. We find that AUTOMAP improves image reconstruction of data acquired on two very different low-field MRI systems: human brain data acquired at 6.5 mT, and plant root data acquired at 47 mT, demonstrating SNR gains above Fourier reconstruction by factors of 1.5- to 4.5-fold, and 3-fold, respectively. In these applications, AUTOMAP outperformed two different contemporary image-based denoising algorithms, and suppressed noise-like spike artifacts in the reconstructed images. The impact of domain-specific training corpora on the reconstruction performance is discussed. The AUTOMAP approach to image reconstruction will enable significant image quality improvements at low-field, especially in highly noise-corrupted environments.

## Introduction

MRI scanners that operate in the low magnetic field regime (i.e., < 0.3 T) as a rule suffer from reduced image quality that arises from the low Boltzmann polarization at these field strengths resulting in weak NMR signals^1^. Accordingly, images obtained at low-field suffer from low signal-to-noise ratio (SNR) which can be mitigated in part by increased acquisition times^2^. The situation is far worse in the militesla regime including efforts at ultra-low field (ULF) (< 10 mT) where, in addition to extremely small NMR signals, spatial encoding gradient field strengths are usually weak (< 10 mT/m), limiting attainable spatial resolutions on these systems compared to those at high field^3^. Despite these challenges, there has been significant recent interest in ULF MRI as a low-cost strategy for increasing the availability and worldwide accessibility of MRI scanners^4^. The ability to acquire images at ULF has been bolstered by contemporary hardware developments including high-performance magnets, RF coils, and gradient sets, and as well as improved strategies for data acquisition and new image processing algorithms^2,5,6^. However, as part of this evolving arsenal of tools, the introduction of recently developed machine learning (ML) approaches has not yet impacted low-field MRI, namely noise-robust machine learning-based image reconstruction.

The application of ML to the problem of transforming the acquired (i.e., raw) data to a final image in a process known as image reconstruction has led to a host of technical improvements including rapid (of order milliseconds) reconstruction of high-dimensional raw data, improvement in image quality and image contrast for highly-undersampled data sets, and dynamic and multi-channel image reconstruction^7–14^. To date, none of these SNR-boosting ML approaches have been applied to real noisy MRI data acquired at very low magnetic field, a regime with unique challenges in acquisition and reconstruction. MR imaging coils must maximize coverage over the volume of interest while minimizing losses, and at low field (i.e., low Larmor frequency), these losses are mainly due to resistive losses in the coil (the so-called Johnson noise regime) as body/sample noise is negligible. RF coil designs using parallel imaging technology are limited in their ability to accelerate imaging at low magnetic field due to a general need to signal average to obtain sufficient SNR; trading acceleration for a loss in SNR is a zero-sum game in terms of acquisition time in this regime. While minimizing losses during signal acquisition will improve image quality and boost SNR, image reconstruction with noise-robust machine-learning approaches have a role to play in high quality low field imaging.

We describe here the capability of deep learning-based image reconstruction approaches to address the low-SNR challenges of low-field MRI. We focus in particular on the use of the AUTOMAP (Automated transform by Manifold Approximation) neural network framework^10^ and describe the SNR boost and image artifact removal that AUTOMAP provides in comparison to other widely-used image denoising techniques. Various deep learning denoising algorithms have substantially gained attention due to their flexible neural network architectures, less need to manually set parameters, and more generalizable denoising problem solving^15–19^. However, these neural networks have primarily been used for denoising in the spatial- and not the signal-domain, and are focused mainly on Gaussian and other idealized noise distributions due to a general dearth of real-world low-field imaging data.

**Figure 1:**
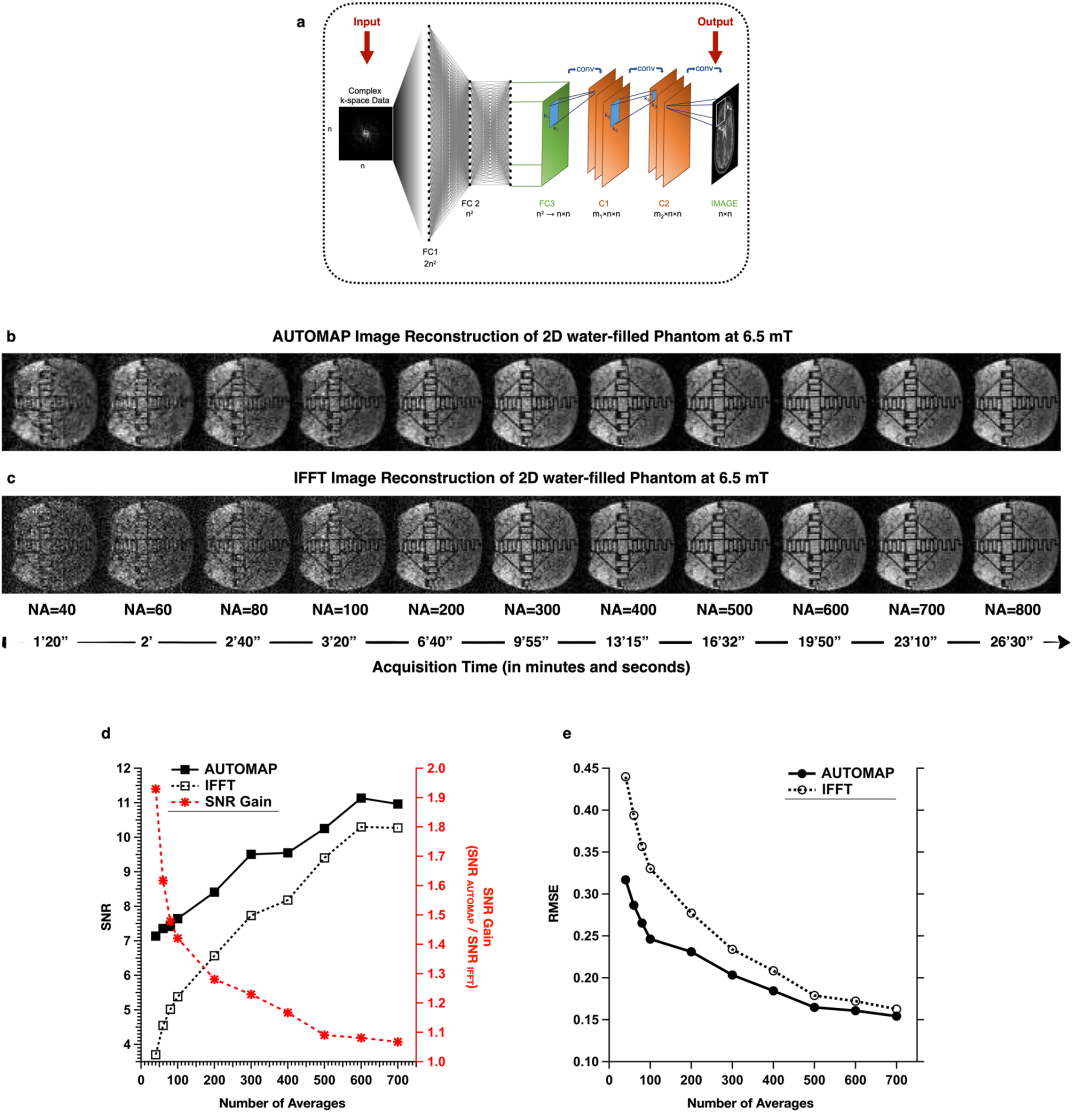
AUTOMAP neural network architecture and AUTOMAP image reconstruction at 6.5 mT—(**a**) the neural network architecture adapted from^10^—the input to the network is the complex *k*-space data and the real and imaginary outputs are combined to form the final image. The network is comprised of two fully connected layers followed by two convolutional layers. (**b**,**c**) Comparison of AUTOMAP reconstruction with IFFT reconstruction for 2D imaging in a water-filled structured phantom—Images were acquired with a bSSFP sequence (matrix size = 64 × 64, TR = 31 ms at 6.5 mT. The number of signal averages (NA) increases from left to right with their respective scan time shown below. (**b**) Upper panel shows AUTOMAP-reconstructed images and (**c**) the lower panel shows same image reconstructed with IFFT. The window level of reconstructed images (**b**,**c** for each NA) is identical. (**d**) SNR Analysis of the 2D phantom dataset as a function of the different number of averages. The mean SNR versus NA is plotted on the left axis for AUTOMAP (filled square) and IFFT (open square). The SNR gain over IFFT is plotted (in red) on the right axis. (**e**) The Root Mean Square Error (RMSE) of the AUTOMAP reconstructed images (filled circle) and the IFFT reconstructed images (open circle) were evaluated with respect to the 800-average IFFT reconstructed image as reference.

The AUTOMAP method recasts image reconstruction as a supervised learning task, whereby the relationship between two data domains, sensor (or *k*-space) and image, emerges during supervised learning from a training corpus generated using the known forward encoding model. Through training, AUTOMAP learns the spatial decoding transform between the *k-*space and the image space. Once trained, the feed-forward network operates between low-dimensional manifolds making the reconstruction highly robust to noise and other artifacts. As described in^10^, the neural network architecture consists of two fully connected layers followed by sparse convolutional layers as shown in Fig. 1a.

Raw data was acquired from two different MRI systems operating at low magnetic field: a 6.5 mT human brain scanner operating in our laboratory^3^ and a 47 mT plant root imager^20^ designed to operate outdoors underground in clay soil. The 6.5 mT ULF scanner was used to acquire image data of both phantoms and human heads. The 47 mT scanner was purpose built to image sorghum root architecture as it grows in natural soils^20^.

In this paper, we assess the reconstruction performance of AUTOMAP on these different low field MRI datasets. For the 6.5 mT scanner, image SNR was evaluated for AUTOMAP reconstructing 2D phantom data and 3D in vivo brain data, as compared to the standard Inverse Fast Fourier Transform (IFFT) reconstruction of the same data. We then compared the SNR boost from using AUTOMAP to jointly denoise and reconstruct the raw data with a compartmentalized approach that first reconstructed with IFFT and then used one of the two state-of-the-art image-domain denoising pipelines—(1) a deep convolutional neural network Gaussian noise denoiser^18^ and (2) the Block-matching and 3D filtering (BM3D) denoising algorithm^21^. The ability of AUTOMAP to remove system imperfections such as noise intrusion from the raw data was also assessed and compared to a denoising pipeline operating in image-domain.

The role of training corpus in the reconstruction performance was then explicitly studied by reconstructing raw data acquired on the 47 mT scanner of sorghum roots with AUTOMAP trained on each of three different training sets: (1) brain images, (2) synthetic vascular tree-like images, and (3) synthetic plant root images. Reconstruction performance was evaluated in each case compared with the standard IFFT reconstruction method.

## Results

### 2D structured phantom MR Imaging at 6.5 mT

In order to characterize image SNR improvement in a well-controlled environment, a phantom experiment was carried out whereby a single slice image was acquired with different number of averages (NA): 40, 60, 80, 100, 200, 300, 400, 500, 600, 700, and 800 respectively.

The 2D water-filled phantom raw datasets were reconstructed with AUTOMAP trained on T_1_-weighted MRI brain images acquired at 3 T. The SNR of the reconstructions was compared with the conventional IFFT reconstruction method. As observed in Fig. 1 b and c, all the slices reconstructed with AUTOMAP exhibit a lower noise and overall image quality is improved such that small features become observable even at low number of averages. The overall mean SNR was evaluated and plotted in Fig. 1d—left axis. The SNR of AUTOMAP reconstructed images were higher than the SNR of IFFT reconstructed images. The mean gain in SNR was of 62% with NA = 40 (Fig. 1d—right axis) and plateaus at NA = 800 with less than 5% gain compared to the IFFT. Taking the reference image as that acquired with the maximum NA, the graph in Fig. 1e shows a significant reduction in the RMSE at lower NA compared to higher NA. The decrease in RMSE for NA less than 200 is 35–40% and 10–20% for higher NA. The error maps (Fig. S1) show a significant decrease in error in the AUTOMAP reconstructed 2D images with respect to the reference IFFT image at NA = 800, compared to the IFFT reconstructed 2D images.

### 3D human brain MR Imaging at 6.5 mT

**Figure 2:**
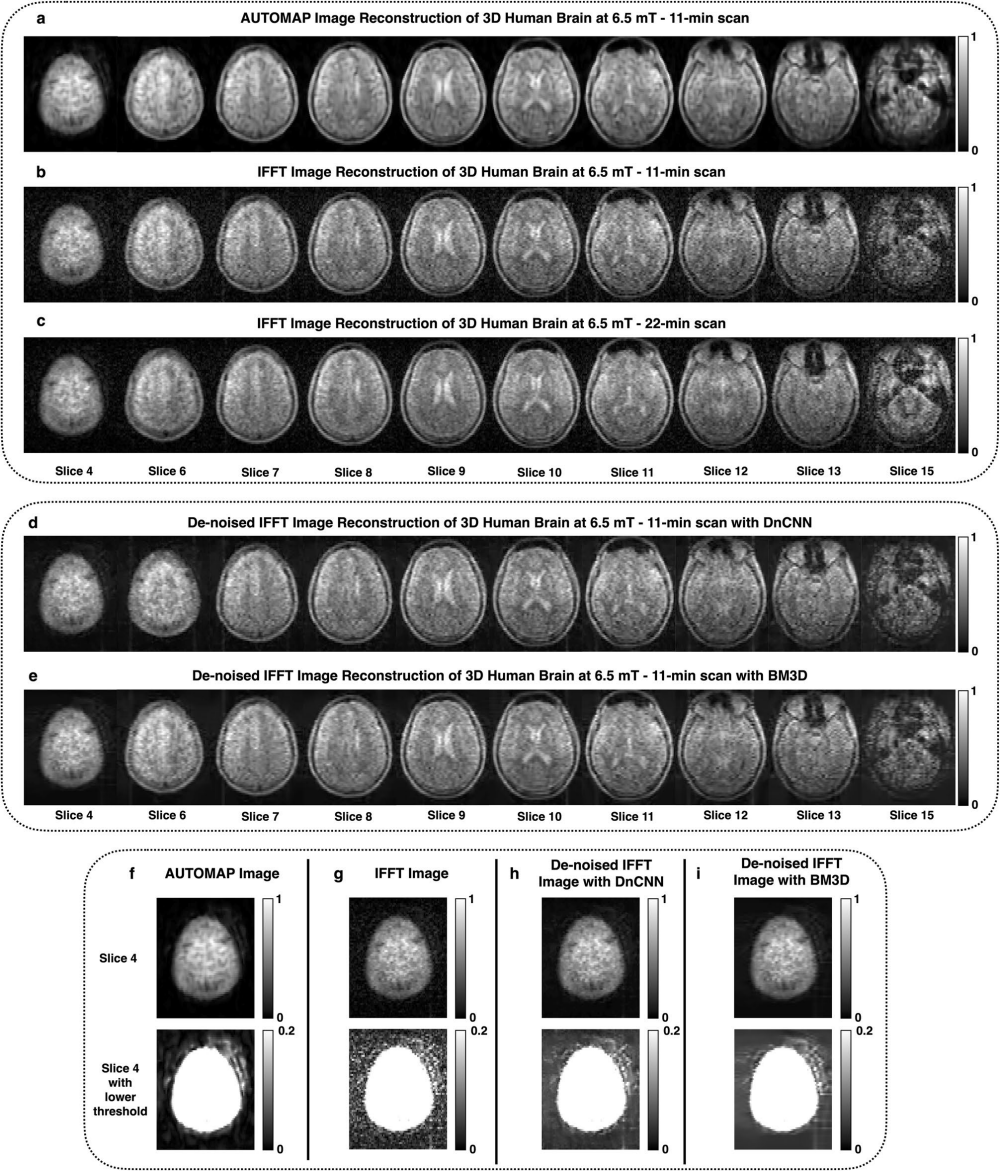
3D brain image reconstruction at 6.5 mT using AUTOMAP compared to conventional IFFT with or without additional image-based denoising pipelines. (**a**–**c**) Reconstruction of 3D human head dataset—an 11-min (NA = 50) 3D acquisition dataset was reconstructed with AUTOMAP (**a**) and IFFT (**b**). Shown here are 10 slices from the full 15 slice dataset. For comparison, a 22-min (NA = 100) acquisition reconstructed with IFFT is shown in (**c**). The window level is unchanged in all images. (**d**,**e**) The two denoising algorithms (DnCNN and BM3D respectively) were applied to the IFFT reconstructed brain image (magnitude only) to compare to the denoising performance of AUTOMAP. (**f**–**i**) Noise floor comparison—Slice 4 from the NA = 50 reconstructed brain dataset shown above in (**a**,**b**) is displayed here with two different window levels: a normalized image on the top and a window level chosen to highlight the noise at the bottom. AUTOMAP is shown in (**f**), and IFFT is shown in (**g**). An additional DnCNN or BM3D image denoising step was applied to the image data reconstructed with IFFT (**h**,**i** respectively).

Healthy volunteer human subjects were scanned under IRB in our 6.5 mT scanner with a 3D-bSSFP sequence. The raw *k*-space data was reconstructed with either AUTOMAP or IFFT. The AUTOMAP reconstructed images of a typical subject acquired with 50 averages in 11 min are shown in Fig. 2a. In comparison with the IFFT reconstruction method (Fig. 2b), the AUTOMAP reconstructed images have lower noise floor and also more clarity can be seen in brain structures such as the ventricles. The reference slices acquired with 100 averages in 22 min are shown in Fig. 2c. Mean SNR improvements in the brain region of 1.5–4.5 times higher were obtained across slices.

As observed in both the phantom and brain datasets, the AUTOMAP approach significantly reduces the noise floor in the reconstructed images as a consequence of operation as a transformation between learned low-dimensional manifolds^10^.

In this light, we wanted to compare the end-to-end reconstruction and denoising performance of AUTOMAP with two different contemporary denoising algorithms. The first denoiser is a state-of-the-art image-only deep learning denoising approach (DnCNN)^18,19^ (recently incorporated as a built-in MATLAB function denoiseImage), which utilizes a deep single-scale convolutional neural network in a residual learning context to perform the denoising. The second denoiser is the well-established BM3D algorithm with collaborative filtering ^21^ (http://www.cs.tut.fi/~foi/GCF-BM3D/bm3d_matlab_package_3.0.7.zip). DnCNN and BM3D were both applied to the (magnitude) images obtained from reconstruction using IFFT, as shown in Fig. 2d and e respectively. A single illustrative slice from the NA = 50 reconstructed brain dataset from Fig. 1g is displayed in Fig. 2f–i, reconstructed with either AUTOMAP (Fig. 2f) or IFFT reconstruction (Fig. 2g). To improve the display of the background noise, the images are shown with the window-level adjusted to more conveniently evaluate the low pixel values. The additional DnCNN or BM3D image denoising step was applied to the image data reconstructed with IFFT (Fig. 2h and i). The denoised IFFT images in Fig. 2d, e, h and i seem to indicate that both DnCNN and BM3D act more on the background and not the signal.

**Figure 3:**
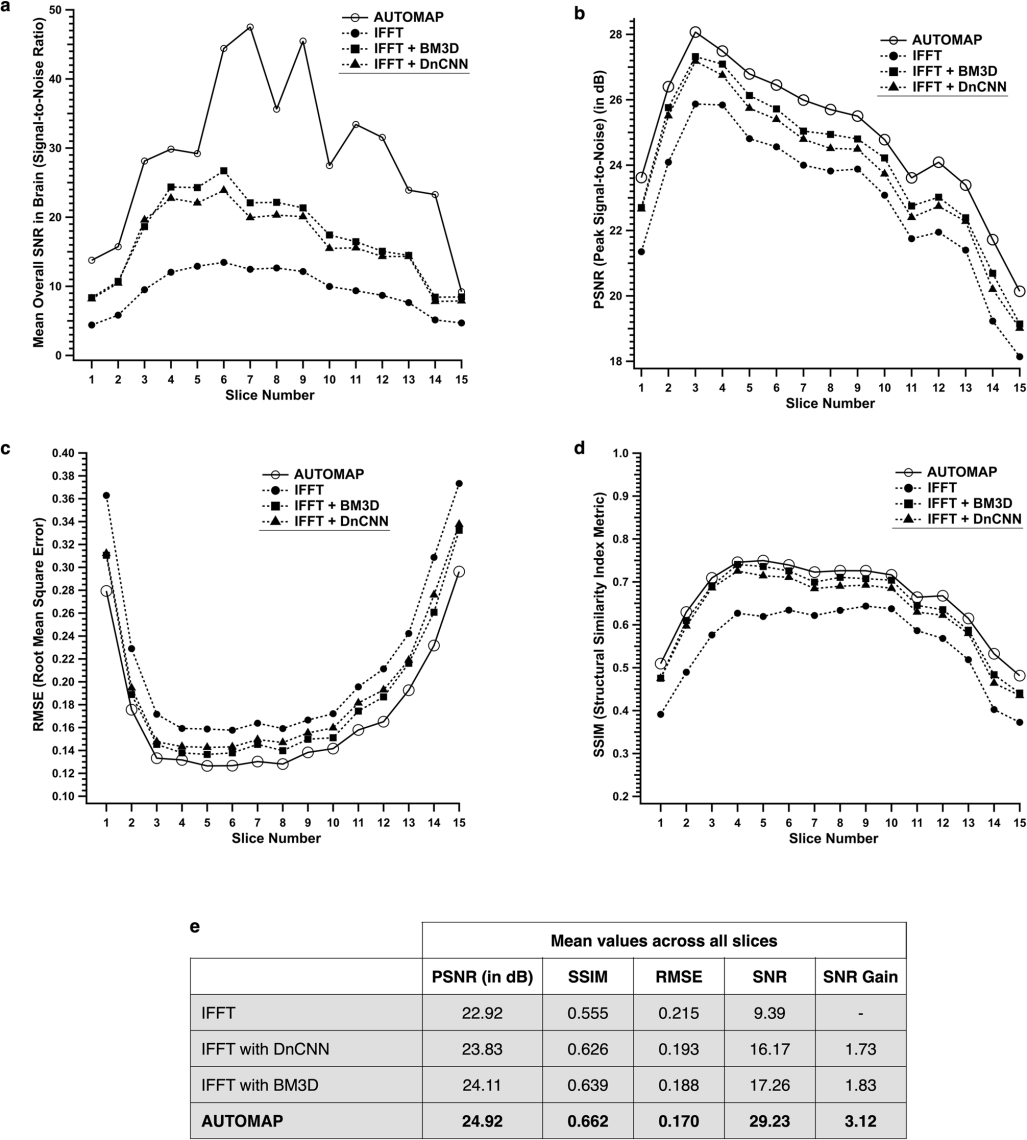
Image metric analysis on the 3D human brain dataset. (**a**–**d**) Image metric analysis of AUTOMAP and IFFT reconstruction with- and without the DnCNN step or the BM3D denoiser following transformation of the raw *k*-space data—The mean overall SNR in the whole-head ROI across all the 15 slices is shown in (**a**) for IFFT (filled circle), denoised IFFT with BM3D (filled square) with DnCNN (filled triangle), AUTOMAP (open circle). Three additional metrics are computed: PSNR (**b**), RMSE (**c**), and SSIM (**d**). (**e**) The table summarizes the mean PSNR, SSIM, RMSE, SNR and SNR gain values across all the slices. The SNR gain was calculated with respect to the conventional IFFT.

The denoised images were evaluated using image quality metrics and compared to those reconstructed without the additional DnCNN/BM3D denoising stage. Results are shown in Fig. 3a–d. The mean overall SNR within the head region is plotted in Fig. 3a. The mean SNR for AUTOMAP is higher by ~ 2 fold than both the IFFT + DnCNN or IFFT + BM3D. The Peak Signal-to-Noise Ratio, PSNR (Fig. 3b) is also ~ 2.0 dB higher for the AUTOMAP as compared to the IFFT alone. The PSNR of IFFT + BM3D is improved by 1.0 dB over IFFT alone; 1.0 dB of the improvement in the PSNR is due to AUTOMAP. The RMSE is lower for AUTOMAP compared to the IFFT + DnCNN or IFFT + BM3D (Fig. 3c). The same conclusion applies for the Structural Similarity Index Metric, SSIM analysis, where the similarity index is higher for images reconstructed with AUTOMAP alone than for those reconstructed via IFFT + DnCNN or IFFT + BM3D images (Fig. 3d). It should be noted that PSNR, SSIM and RMSE metrics rely on the reference head scan with 100 averages which is only twice the NA and can be considered as a possible limitation. This imaging and analysis were repeated for a second human subject and similar results were obtained (data not shown). The table in Fig. 3e summarizes the mean metric values across all the 15 slices and shows that AUTOMAP alone provides a mean SNR gain of 3.1 with a maximum at 4.5.

**Figure 4:**
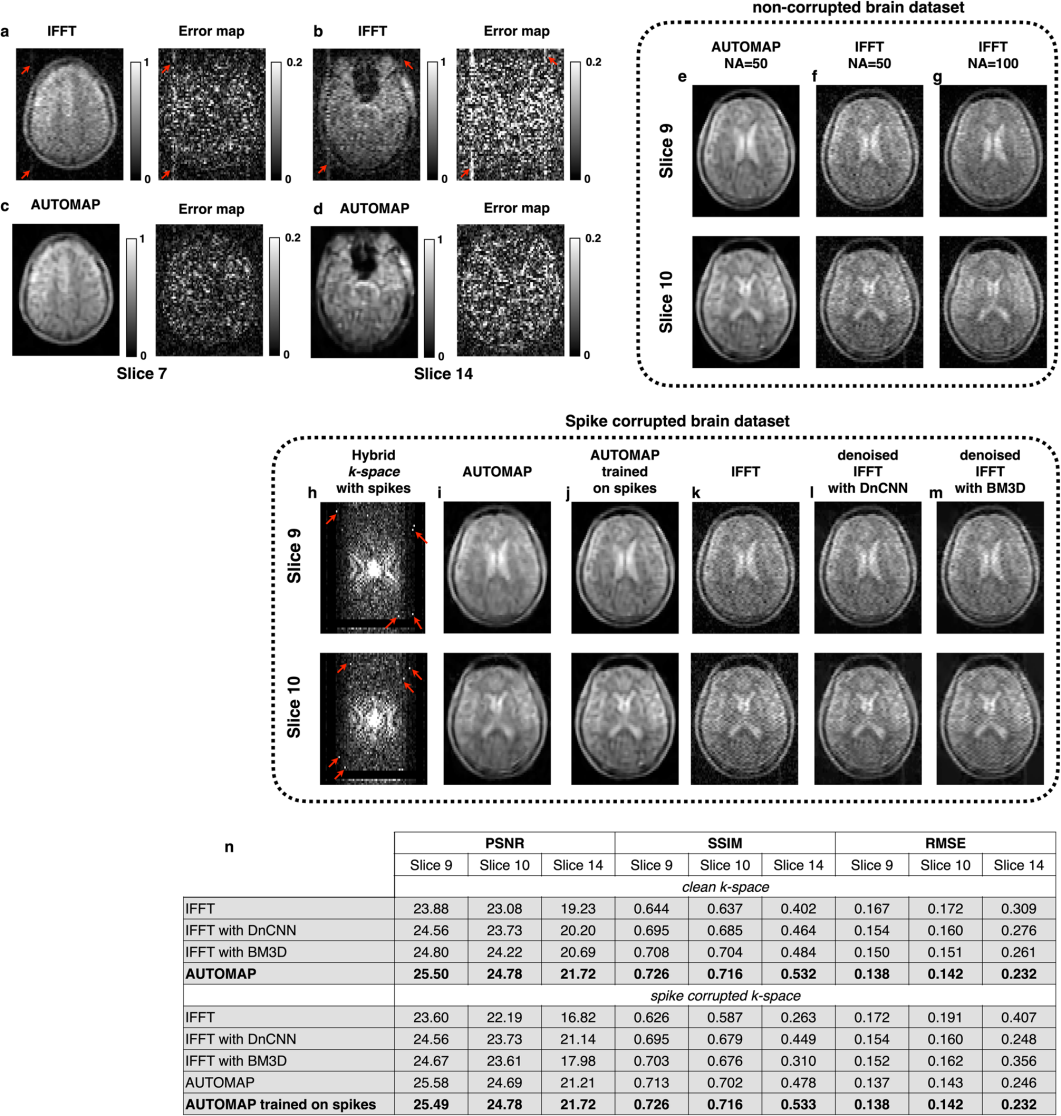
Artifacts: (**a**–**d**) Elimination of hardware artifacts at 6.5 mT—Two slices from a 3D bSSFP (NA = 50) are shown. When reconstructed with IFFT (**a**,**b**), a vertical artifact (red arrows) is present across slices. When the same raw data was reconstructed with AUTOMAP (**c**,**d**), the artifacts are eliminated. The error maps of each slice with respect to a reference scan (NA = 100) is shown for both IFFT and AUTOMAP reconstruction. (**e**–**g)** Uncorrupted *k*-space (NA = 50) was reconstructed with AUTOMAP (**e**) and IFFT (**f**). The reference NA = 100 scan is shown in (**g**). **(h**–**m**) AUTOMAP reconstruction of simulated *k*-space artifacts. Two slices of the hybrid *k*-space from the 11-min (NA = 50) brain scan was corrupted with simulated spikes (**h**). In (**i**), the data was reconstructed with AUTOMAP trained on the standard corpus of white Gaussian noise corrupted brain MRI images. In (**j**), the *k*-space data was reconstructed with AUTOMAP with a training corpus of *k*-space data including variable number of random spikes. IFFT reconstructed images are shown in (**k**), where the spiking artifacts are clearly seen. Denoised IFFT with DnCNN reconstructed images are shown in (**I**) and denoised IFFT with BM3D reconstructed images are shown in (**m**). (**n**) The table summarizes image quality metrics for the reconstruction task of the three slices, both with- and without spike corruption. PSNR, SSIM and RMSE were evaluated for reconstruction using IFFT, denoised IFFT with either DnCNN or BM3D, and AUTOMAP trained on either the standard Gaussian noise-corrupted corpus or on a spike- and Gaussian noise-corrupted corpus.

In addition to the improvement in SNR and robustness to noise, the use of AUTOMAP has the potential to reduce or eliminate certain imaging artifacts (see Fig. 4a–d) which might arise from a low-field scanner operating in an unshielded environment or other real-world non-ideal imaging scenarios. A human head scan acquired at 6.5 mT in 22 min (NA = 50) in our laboratory (Fig. 4a) inadvertently contained a spiking artifact caused by noisy gradient amplifier switching that can be seen as structured noise in the phase-encode direction across all slices when reconstructed with IFFT. A reference scan (NA = 100) was acquired with the scanner operating normally (i.e., with no artifact), and error maps between the reference and the NA = 50 scan also depict clearly the spike artifact in Fig. 4a and b. This artifact was eliminated when the same dataset was reconstructed with AUTOMAP as seen in Fig. 4c and d.

As a way to evaluate the ability of AUTOMAP to reduce *k*-space spike artifacts, we randomly generated high-intensity *k-*space samples, which were added to the hybrid *k-*space data to simulate spike corrupted multi-slice *k*-space data (Fig. 4h). This data was reconstructed with the same trained model used earlier (see “Methods” for details). As described in^10^, the AUTOMAP reconstruction approach may be seen as a transformation between manifolds that are conditioned during training. In this case, since the training corpus was based on clean, artifact-free brain MRI data, spike artifacts would be detected as non-signal noise-like features outside the training corpus and be suppressed during the reconstruction. The AUTOMAP reconstruction of the spike corrupted *k*-space (Fig. 4i) is mostly artifact-free compared to the highly-corrupted images as can be seen in the IFFT reconstruction (Fig. 4k), although there are still some residual artifact-like structures as quantified in the table described in Fig. 4n. We note that the non-corrupted images reconstructed with IFFT are shown in Fig. 4f and g.

To further improve the immunity of the learned approach to spiking artifacts, we trained AUTOMAP on a new training corpus that included random spike corruption as described in “Methods”. The spike-corrupted *k*-space data (Fig. 4h) was reconstructed with this new trained AUTOMAP and as seen in Fig. 4j, and the reconstructed images are artifact-free, appearing identical to the uncorrupted *k-*space ground truth reconstructed with AUTOMAP (Fig. 4e). We found that by training AUTOMAP on a corpus that includes forward encoding of classes of corruption that are representative of those likely to be found in the experimental data, reconstruction performance is greatly improved, reducing or even eliminating artifacts in the final image.

A sparse transform that is conditioned on properties of its training corpus operates very differently from a purely image-based denoising method. As shown in Fig. 4l and m, the DnCNN denoising and the BM3D denoising applied following the transform from *k*-space to image-space are not able to significantly reduce spike artifacts beyond blurring them. Quantitative image quality metrics measurements (in Fig. 4n) corroborate this. In particular, the SSIM values of the spike-corrupted dataset reconstructed using the standard Gaussian noise-trained AUTOMAP are slightly lower than the SSIM values of the clean dataset reconstructed using the same model. The SSIM metrics of the spike-corrupted dataset reconstructed using the spike- and Gaussian-noise trained AUTOMAP model are identical to the spike-free dataset reconstructed with the standard Gaussian noise-trained AUTOMAP. In summary, AUTOMAP was able to eliminate most spiking artifacts when trained on a standard corpus without prior knowledge of artifacts, and once trained on a corpus that included spike-like signals, AUTOMAP completely removed spike artifacts with a reconstruction performance equal to that of the uncorrupted data set.

### Random noise stability test on the AUTOMAP neural network

**Figure 5:**
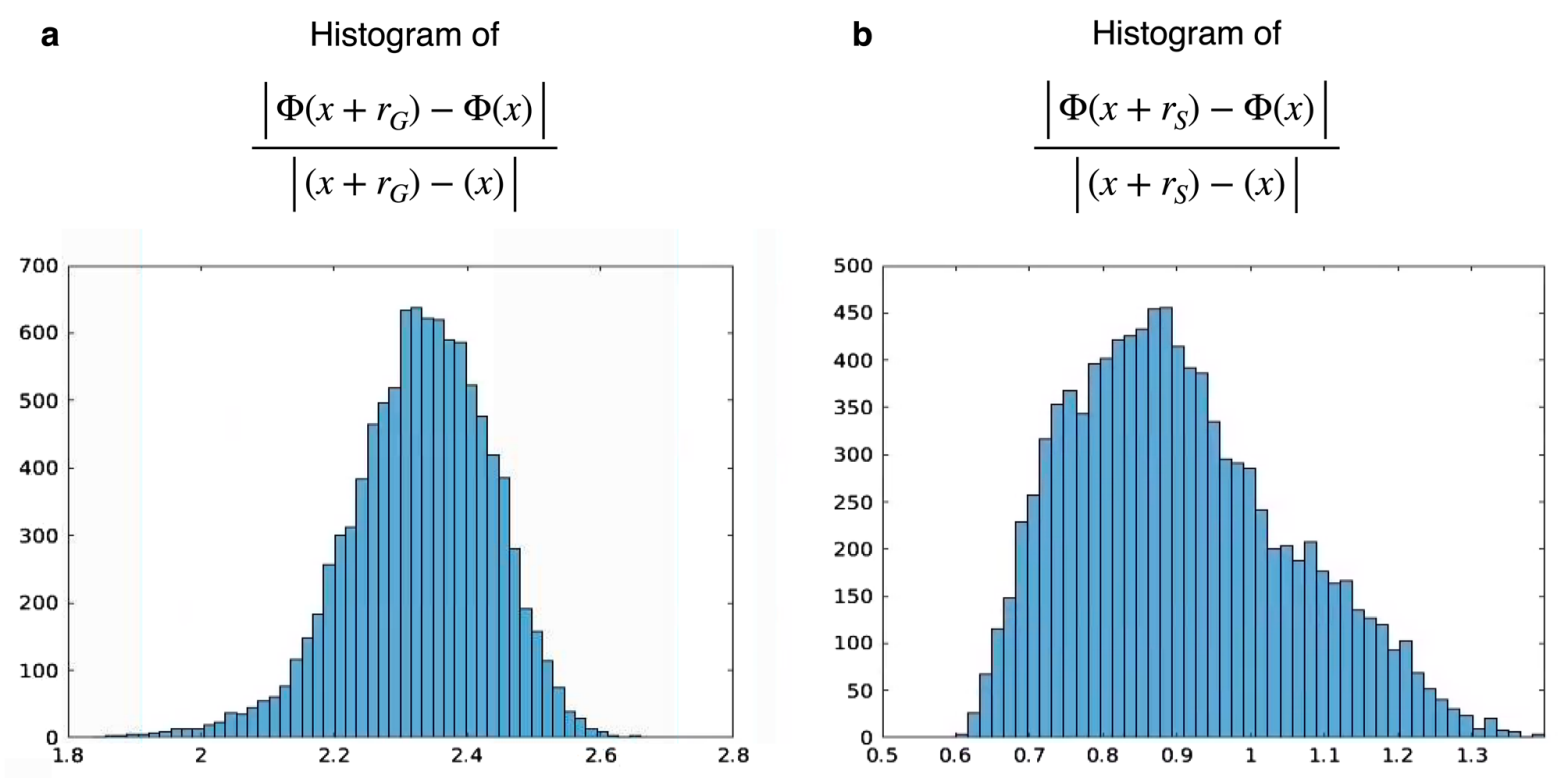
AUTOMAP is locally stable to noise: (**a**) histogram of the output-to-input variation ratio taken over noiseless- and Gaussian noise applied input data, with a low maximum value of 2.7. (**b**) histogram of the same output-to-input variation ratio taken over noiseless and Gaussian plus spike noise applied to the input data, with low maximum value of 1.5.

In order to understand how stable the AUTOMAP reconstruction process to the presence of realistic noise, we performed a noise stability test of the neural network used above. The local robustness of the Gaussian noise AUTOMAP trained neural network was computed empirically on the maximum ratio between variations in the output space and variations in the input space: $\frac{{\left| {{\Phi }\left( {x^{\prime}} \right) - {\Phi }\left( x \right)} \right|}}{{\left| {{ }x^{\prime} - x} \right|}}$ for separate images *x* and *x*′. 10,000 pairs of HCP brain images were used for this experiment. The first set of 10,000 images were used as *x* and their corresponding *k*-space data was generated. For the second set of 10,000 images, *x*′, 15–35 dB of additive white Gaussian noise was randomly added on their corresponding *k*-space data. The phase images of the brain images were kept the same between the pairs of brain images. Both *k*-space data were reconstructed with the noise-corrupted trained model. A maximum output-to-input variation ratio of 2.7 is observed for the Gaussian noise ranging from 15 to 35 dB as shown in Fig. 5a.

The same experiment was carried out with the spike- and noise- AUTOMAP trained neural network. The first set of 10,000 images, *x* and their corresponding *k*-space data were the same as the first experiment. For the second set of 10,000 images, *x*′, random additive Gaussian noise varying from 15 to 35 dB and between 1 and 25 spikes (randomly chosen) were added on their corresponding *k*-space data. The phase images of the brain images were kept the same between the pairs of brain images. A maximum output-to-input variation ratios of 1.5 was obtained. The histogram of these metrics is plotted in Fig. 5b.

These results empirically demonstrate the local stability of AUTOMAP reconstruction against stochastic perturbations.

### Plant root MR imaging at 47 mT

**Figure 6:**
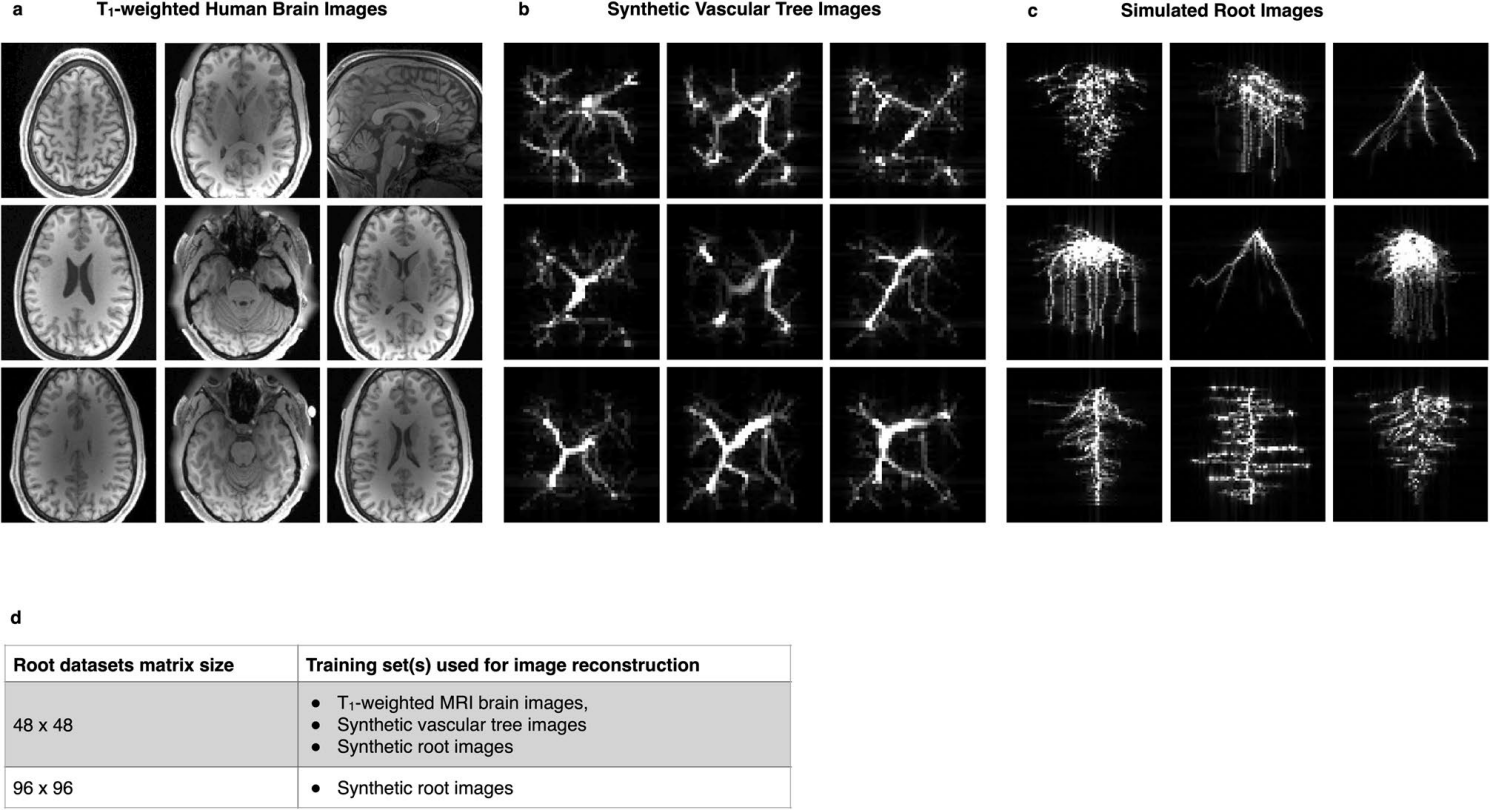
Domain-specific training corpora used on plant roots dataset—(**a**–**c**) Representative images from three training sets for root MRI reconstruction. (**a**) 2D images from the Human Connectome Project database. (**b**) 2D images from the training set based on synthetic vascular trees. Each of the 2D images was obtained by summing up the 3D synthetic vascular tree volumes in all 3 dimensions. (**c**) Images of realistic simulated root system from the RootBox toolbox. (**d**) The list of matrix sizes of the acquired root datasets and their corresponding training set used for image reconstruction.

Recent prior work has demonstrated the feasibility of studying the root phenotype of sorghum growing in natural clay soils with low field MRI^20^. With the aim of further improving root images acquired from the 47 mT cylindrical electromagnet-based scanner described therein, raw *k*-space from several 2D root projection imaging experiments were reconstructed with AUTOMAP and the images were compared with the conventional IFFT reconstruction. In addition to evaluating the performance of AUTOMAP to reconstruct root images compared to IFFT, we also investigate use of different training corpora (Fig. 6a–c) and the impact of each training corpus on the reconstructed images. Figure 6d summarizes the list of the different resolution of the acquired root datasets and the corresponding training set used for reconstruction.

**Figure 7:**
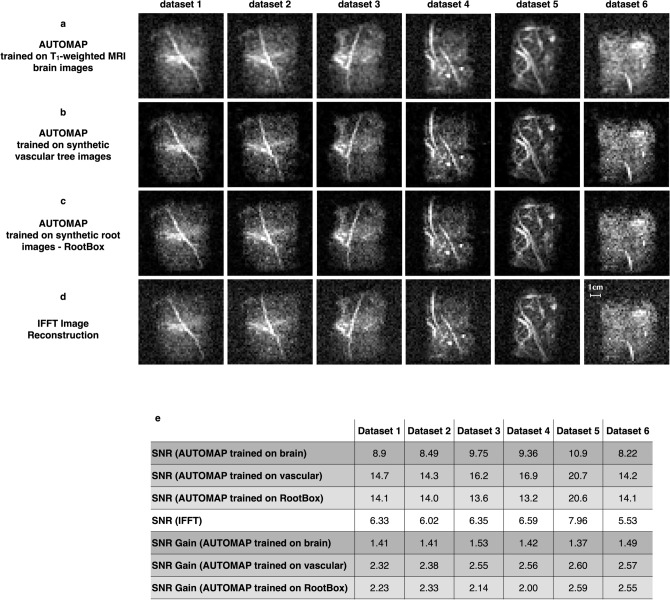
Reconstruction of sorghum dataset acquired at 47 mT using AUTOMAP trained either on T_1_-weighted MRI brain images, synthetic vascular tree Images, or synthetic root images. Six 2D projections of roots images (labelled dataset 1–6) extracted from six different roots’ samples are shown. All the 48 × 48 datasets were acquired at 47 mT. In the upper panel (**a**), the raw data were reconstructed with AUTOMAP trained on T_1_-weighted MRI brain images, the panel (**b**) are the same datasets reconstructed with AUTOMAP trained on synthetic Vascular Tree Images and finally the same datasets were reconstructed with AUTOMAP trained on synthetic root images in panel (**c**). The lower panel (**d**) shows the images reconstructed with the standard IFFT method. All the images were windowed to the same level for comparison. The scale of 1 cm represented one of the images is the same for all the 2D projections. (**e**) The table summarizes the mean SNR analysis of the 2D projections of the roots 48 × 48 datasets acquired at 47 mT.

First, 48 × 48 root *k*-space datasets were reconstructed using the “brain trained” model, i.e., where the training set was composed of T_1_-weighted MRI brain images that were downsampled to match the 48 × 48 matrix size. The results of the reconstructed 2D data in Fig. 7a show consistent improvement in SNR with AUTOMAP across all the datasets. AUTOMAP was able to reconstruct the images accurately, without any visible distortion or information loss as the standard IFFT images as seen in Fig. 7d, and with better overall SNR of the roots. Enhancements of 1.37–1.53 were observed in the different images as stated in the Fig. 7e. The noise floor in all AUTOMAP reconstructed datasets are considerably reduced.

We note here that by visual inspection, the sulcus-like structures in the MRI brain image data (see Fig. 6a) are not present in the linear and branching features of the root images. As a first attempt to generate a more domain-appropriate training corpus for roots that would enhance feature extraction and improve the image quality of the root reconstruction, we naïvely drew visual comparison to the natural branched structure of blood vessels, and leveraged existing synthetic vascular tree models to generate training images (Fig. 6b) This vascular tree-based trained model is described in the Methods and was evaluated by measuring the mean overall SNR of the images and comparing them with the IFFT method. The results are shown in Fig. 7b, where a noticeable reduction in noise is seen in all the six root datasets. The SNR enhancement evaluated in those images are reported in the Fig. 7e. A further improvement in SNR was obtained with SNR gains of 2.3- to 2.6-fold higher than the IFFT reconstruction. Along with the decrease in noise floor, we also observed that the small signal arising from soil water is attenuated. This filtering of soil signal can eventually help in extracting specific root measurements using existing root data analysis toolbox.

Our third training corpus for root reconstruction uses a specialized root architecture toolbox—*RootBox*—to generate synthetic root structures, and this was used to train AUTOMAP (https://github.com/Plant-Root-Soil-Interactions-Modelling/CRootBox). Some representative root reconstructions are shown in Fig. 6c, and these reconstructed images in Fig. 7c appear to be nearly indistinguishable from those reconstructed with vascular tree-based model. Figure 7e summarizes the results as SNR and SNR enhancements for all three trained models and clearly shows that the domain specificity of the synthetic vascular tree and the synthetic roots promotes the reduction in the noise floor in the images and takes advantage of the sparse features of those two training sets.

**Figure 8:**
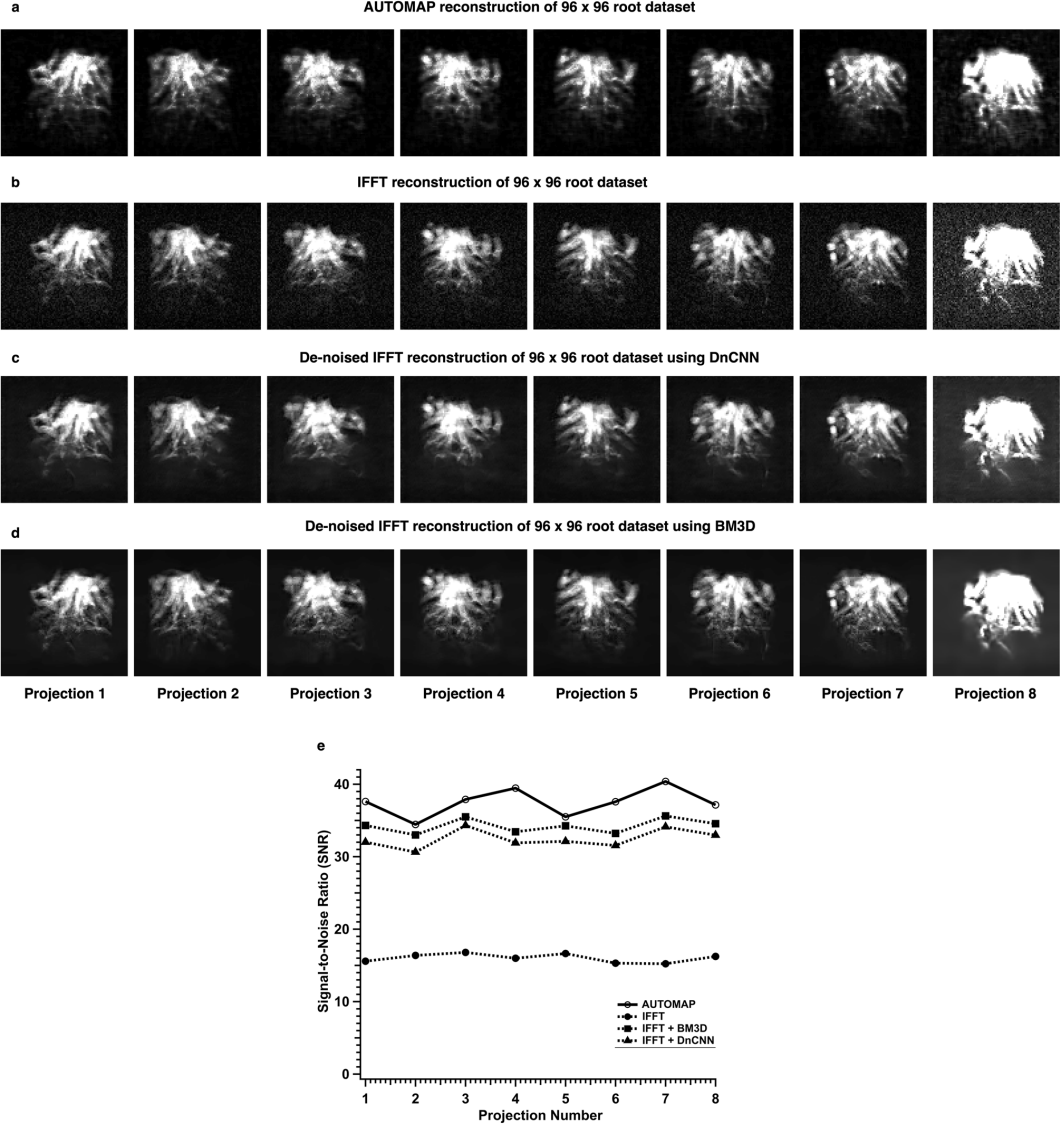
AUTOMAP reconstruction using the RootBox synthetic roots database versus IFFT reconstruction of a 96 × 96 root dataset. All eight 2D projections reconstructed with AUTOMAP are shown in (**a**), and with IFFT in (**b**). Each of the magnitude image was processed with either DnCNN as shown in the third panel (**c**) or with BM3D in the fourth panel (**d**) The window level for projections 1–7 were set to the same value except for projection 8, where the threshold was lowered on both panels to reveal the noise floor differences. To generate figure (**e**), the SNR was evaluated for AUTOMAP reconstruction using the RootBox training and compared to IFFT reconstruction with and without the denoising pipelines, and the SNR of each of the 8 projections reconstructed with AUTOMAP (open circle), IFFT without the denoising algorithm (filled circle), IFFT with BM3D (filled square), and IFFT with DnCNN (filled triangle) is plotted.

Roots datasets acquired with a higher spatial resolution of 0.83 mm were also reconstructed using synthetic root trained AUTOMAP model since the roots appeared to be more densely packed root crown structures. All the eight projections from a root dataset were reconstructed with AUTOMAP and a significant reduction in noise floor can be observed in Fig. 8a as compared with IFFT in Fig. 8b. The magnitude root images reconstructed with IFFT were then processed with the two denoising pipelines—DnCNN and BM3D as shown in Fig. 8c and d respectively. SNR improvements with AUTOMAP reconstruction of 2-fold or higher than IFFT were observed. AUTOMAP reconstruction of the roots was also slightly higher than denoised IFFT with BM3D or DnCNN. Figure 8e summarizes the SNR analysis of both the AUTOMAP reconstruction and the IFFT reconstruction over the eight projections.

## Discussion

In this work, we have shown that our end-to-end deep learning-based image reconstruction approach improves reconstruction of SNR-starved MRI images acquired at low magnetic field. From the structured phantom images and human brain images acquired at 6.5 mT, to plant root images acquired at 47 mT, we observed significantly improved image SNR in all cases with AUTOMAP reconstruction. The well-established relationship between SNR and scan time allows us to utilize the gain in SNR from AUTOMAP to enable a reduction in scan time of at least twofold. Indeed, the results from the 2D structured phantom shows that as the number of averages decreases, the AUTOMAP reconstruction approach is able to extract more features from the input *k*-space as compared to the conventional IFFT method. Also, we should note that the reconstruction of the phantom data was done using a model that was trained on T_1_-weighted brain images acquired on a 3 T scanner. Even though brain structures exhibit different visual features than the phantom, the model learned the relationship between the *k-*space and the image and reconstructed the data with improved SNR and also shows robustness to noise^10^.

Furthermore, in the in vivo human experiment, training the neural network without the addition of white Gaussian noise in the *k-*space shows mean SNR improvements of 1.3 to 1.54-fold corresponding to 1.0 to 1.5 dB increase in the PSNR (data not shown). However, the addition of random white Gaussian noise (15–35 dB) in each *k-*space data in the training database shows a further improvement in the output images with significant noise reduction. As per the SSIM values, we did not observe any loss of information compared to the reference image dataset.

State-of-the-art denoising methods like BM3D^17^, WNNM^22^ or TNRD^23^ which are based on nonlocal self-similarity image prior models are popular and effective denoisers that generally act on the reconstructed magnitude images. However, these approaches typically employ time-consuming iterative optimizations that suffer from generalization to multiple noise levels. Zhang et al. proposed a deep convolutional neural network denoising (DnCNN) approach that uses a residual network architecture to learn the noise and subtract it from the image. This work showed that the state-of-the BM3D with collaborative filtering as well as the DnCNN approach can attenuate the additive white Gaussian noise operating purely on the image (magnitude) data for a range of noise levels, and that DnCNN has been evaluated as one of the standard machine learning methods to denoising magnitude MR images^19^. We compared DnCNN-denoised low field images and BM3D-denoised low field images to AUTOMAP reconstructed images, in particular on the in vivo brain dataset. Because BM3D and DnCNN are both limited to reducing noise only in the image domain, its reconstructed images generally exhibited over-blurred features. We also note that the statistical properties of the noise profile changed after denoising. On the contrary, AUTOMAP, which operates directly on the raw data to solve the inverse problem operating between learned low-dimensional manifolds, is more robust to noise at a fundamental level. From the quantitative analysis, we see that AUTOMAP reconstructed images have a higher PSNR and SSIM values than denoised IFFT images.

Reconstructing the raw *k*-space data using AUTOMAP has another interesting advantage in cases involving high frequency spike-corrupted datasets. The reconstruction of spike-corrupted data demonstrated significant suppression of artifacts; these were outside the training corpus and thus treated as non-*k-*space-like data and were supressed by the transform and essentially eliminated from the reconstructed image. In addition, we observed significant improvement in image accuracy with improved SSIM values as compared with the denoising pipeline.

As mentioned above, both of the image-based denoising pipelines that we tested reduce Gaussian noise in the image domain, but also blurs the entire image^24^. On the contrary, AUTOMAP learns that spike artifacts in the sensor domain do not contain any ‘signal-like’ or “image-like’ information and hence tries to discard them. Furthermore, if training is performed on an artifact-based database, where the neural network learns the relationship between a corrupted *k*-space and a clean image, we show that the SSIM, the PSNR and the RMSE values of the reconstructed images with corrupted *k*-space match those images with clean *k*-space data. We also note that the artifact-based trained model works better for high spatial frequency spikes. Besides threshold-based spike correction techniques, the Fourier-Transform based approach developed by Yi-Hsuan Kao and his team is an effective spike removal algorithm even for low spatial frequencies^25^. However, some conditions need to be fulfilled for optimal results, for example, 2 spikes have to be at least 7 pixels apart. Another spike removal technique, the Principal Component Analysis algorithm-based approach that acts directly on the *k*-space data, separating the sparse low-rank spike information, is one that successfully identifies high frequency RF spikes efficiently. However, the sparsity penalty parameter needs to be adjusted for every dataset for optimal results^26,27^. Using residual learning can be one of the solutions where the neural network learns the relationship between the corrupted *k*-space and the subtracted image between the clean and corrupted one^28,29^.

The introduction of realistic sources of noise in the training model did not significantly impact image reconstruction. The Gaussian-based trained model was able to reconstruct non-corrupted low-field brain images. The artifact-based trained model was able to reconstruct both non-corrupted and spike-corrupted brain data, demonstrating the stability of the trained model to spike artifacts. Furthermore, the noise stability results over 10,000 images show that the trained neural networks were robust to the types of the noise that were included in their training set. Recent publications have also shown similar robustness of trained neural networks to random input perturbations^30–32^. When applying these reconstruction approaches to critical applications, the local robustness of neural networks should be evaluated for the kinds of real-world noise source that arise in the corresponding physical imaging scenarios. These experimental sources of noise are unlikely to have the unique statistical properties of highly-engineered adversarial noise designed specifically to break the reconstruction process^33^, which in practice has near-zero chance of occurring in physical imaging systems.

Plant root imaging in natural soils (> 10% clay) has proven to be difficult at high field strengths (> 1 T) because the presence of magnetic material in clay soil results in image artifacts and distortions^34,35^. Imaging at low field strengths has been shown to reduce or eliminate image distortions^20^. Another advantage of low field is the feasibility of visualizing the root system architecture by directly measuring the plant roots in the field. However, low field imaging implies poor SNR. Even if with 98% of a typical plant root accounts for water, low field imaging of a few roots of less than 4 mm in diameter each can be challenging. We have shown that the T_1_-weighted HCP brain trained AUTOMAP model was able to further improve SNR and the contrast of the root images compared to the standard IFFT reconstruction. It should be noted that even if the pulse sequence parameters heavily suppress the soil water signals (T_2_ < 4 ms) and not root water signal (T_2_ ~ 100 ms), a strong background signal was observed in the 48 × 48 datasets compared to the 96 × 96 dataset. The presence or absence of the background signal can be related to the magnetic soil content and the soil water content^35^.

Besides training on T_1_-weighted brain images, we investigated domain-specific training on plant root MRI datasets acquired on a 47 mT magnet^20^. Reconstruction of the plant roots datasets is feasible with a T_1_-weighted brain trained model; however, the low-dimensional representation of plant root images is different than that of brain images. The use of a model trained on either vascular tree structures or synthetic roots images provided better SNR improvements. The performance with both training sets were similar and qualitatively the AUTOMAP reconstructed images look the same as the IFFT reconstructed images. Unfortunately, due to lack of ground truth images, SSIM and PSNR were not evaluated. Scaling up AUTOMAP reconstruction to 96 × 96 roots dataset clearly shows the robustness to noise even in conditions where we have higher SNR. The significant improvement in SNR will eventually help in feature extraction of the roots such as the angle of the roots, the number of roots, the length and diameter of the roots. The difference between root structures and noise is enhanced and thereby postprocessing analyses such as segmentation would work more effectively. Imaging roots at low field is feasible, however for characterizing the root phenotype, images with higher spatial resolution are required. Ongoing work is being conducted with the goal to visualize and assess finer roots of less than 1 mm in diameter.

In conclusion, we have applied the end-to-end AUTOMAP ML-based image reconstruction approach to different low field MRI datasets acquired in the mT regime and showed the significant improvement in SNR and image quality without any loss of information. We have demonstrated application to spike-corrupted data and have shown the impact of the training set on the reconstruction. As low field MRI is rapidly becoming a modality of interest for more sustainable and affordable medical care^36^, we believe that employing noise-robust machine learning image reconstruction approaches will be an important component in shortening scan times and improving image quality to accelerate the practical use and adoption of low-field MRI.

## Materials and methods

### 2D phantom: data acquisition at 6.5 mT

2D data at 6.5 mT (276 kHz ^1^H frequency) was acquired with a balanced Steady State Free Precession (b-SSFP) sequence of a hemispheric water-filled structured resolution phantom placed inside a single-channel spiral volume head coil described previously^3^. The sequence parameters were TR = 31 ms, matrix size = 64 × 64, spatial resolution = 4.2 mm × 3.6 mm, and slice thickness = 12 mm. Several imaging sequences were run, each with a different number of averages (NA): 40, 60, 80, 100, 200, 300, 400, 500, 600, 700, and 800 respectively. The total acquisition time of each dataset is shown in Fig. 1b,c.

### 3D in vivo brain: data acquisition at 6.5 mT

The same single-channel spiral volume head coil described above was used to acquired 3D human brain data. A 3D b-SSFP sequence was used: TR = 31 ms, matrix size = 64 × 75 × 15, spatial resolution = 2.5 mm × 3.5 mm × 8 mm, and 50% under-sampled was performed along both along the phase-encode and the slice direction. Two in vivo datasets were collected: (1) a 11-min scan with NA = 50 and (2) a 22-min scan with NA = 100. The 22-min scan was used as the reference scan. The MRI subjects were 2 healthy volunteers. Subjects of age 24–35 were recruited by investigators at Massachusetts General Hospital. There were no self-selection biases or other biases. Informed consent was obtained from each healthy human volunteer prior to the experiment in accordance with the Human Research Committee of the Massachusetts General Hospital (MGH). All MRI imaging was performed in accordance with approved guidelines and regulations, using experimental protocols that were approved by the MGH Human Research Committee.

To simulate spike/herringbone corrupted *k-*space data, we altered the raw *k*-space data of the 11-min scan by multiplying the signal with a scaling factor. The scaling factor varied from 2 to 20 and the number of spikes, which were randomly spread over the *k*-space ranges from 2 to 10.

### Sorghum root imaging at 47 mT

2D projection images were acquired from rhizotron cores^20^. The 2D projection images were acquired using a 2D spin-warp pulse sequence. Sixteen spin echoes with an echo spacing of 7-ms were generated and the image acquisition time for each 2D projection in this experiment was 15 min. The sequence parameters are as follows: TR = 500 ms, field-of-view = 80 mm, matrix size = 48 × 48, and spatial in-plane resolution = 1.64 mm. Different roots data with matrix size of 96 × 96 and spatial resolution of 0.83 mm were acquired for visualizing finer root structures.

### AUTOMAP training dataset for phantom and brain imaging at 6.5 mT

Two training corpora were assembled from 51,000 2D T_1_-weighted brain MR images acquired at 3 T selected from the MGH-USC Human Connectome Project (HCP) public database^37^. 135 subjects were used to generate the database. Each subject folder contained 3D T_1_-weighted magnitude only images of matrix size 256 × 320 × 320 which were first cropped to 256 × 256 × 256. For each anatomical plane (coronal, sagittal or transverse), only the middle 128 slices were selected and then subsampled to either 64 × 64 (for the phantom dataset) or 75 × 64 (for the brain dataset), symmetrically tiled to create translational invariance. Random additive white Gaussian noise (ranging from 15 to 35 dB) was applied to each image in the training set. To produce the corresponding *k-*space for training, each image was Fourier Transformed with MATLAB’s native 2D FFT function. The neural network was trained from the noise corrupted *k-*space encodings and the target cropped, subsampled HCP brain images to learn an optimal feed-forward reconstruction of *k-*space domain into the image domain.

### Spike/herringbone artifact AUTOMAP training dataset for brain imaging at 6.5 mT

An artifact-based 75 × 64 training set was generated using the same HCP dataset. The *k*-space inputs were corrupted with randomly distributed spikes outside of the central 17% of *k*-space. The number of spikes varied from 1 to 25 and its magnitude was multiplied randomly by a factor between 2 and 30. Additive white Gaussian noise was also added in the *k*-space data (ranging from 15 to 35 dB). The neural network was hence trained from the noise corrupted spiky *k-*space encodings and target ‘noise-free’ images using a neural network architecture as described below.

### AUTOMAP training dataset for sorghum imaging at 47 mT

AUTOMAP was trained on the Fourier forward-encoding model using three different training corpora and each model was used to reconstruct the low-field plant root raw data. The performance of AUTOMAP on those three training sets was evaluated.

Brain training set—the first training set used was built from the same HCP database as mentioned above, using 51,000 2D T_1_-weighted MRI brain images (shown in Fig. 6a) downsampled to matrix size 48 × 48. The images in each training set were symmetrically tiled to create translational invariance and finally normalized to the maximum intensity of the data. To produce the corresponding *k-*space representations for training, each image was Fourier Transformed with MATLAB’s native 2D FFT function.

Vascular training set—the second training set was assembled from 55,000 2D synthetic vasculature images using the Vascular Tree Synthesis Software, VascuSynth^38^. The first step was to generate 3D volumes of the random synthetic vessels that were 48 × 48 × 48 with random branching sizes, numbers and flow rates. Then 2D images of the vasculature were obtained by randomly slicing the volumes and summing in either the first, the second or the third dimension, resulting in 55,000 images in total. Examples from the training set are illustrated in Fig. 6b. Random additive white Gaussian noise (ranging from 10 to 35 dB) was applied to each image in the training set. Each image was symmetrically tiled to create translational invariance and finally normalized to the maximum intensity of the data. To produce the corresponding *k-*space representations for training, each image was Fourier Transformed with MATLAB’s native 2D FFT function. The neural network was trained from the noise corrupted *k-*space encodings and target ‘noise-free’ images to learn an optimal feed-forward reconstruction of *k-*space domain into the image domain.

RootBox training set—the third corpus was assembled from 75,000 2D synthetic 48 × 48 root images. The roots images were generated using a 3D root system growth model implemented with MATLAB—called RootBox^39^ (see Fig. 6c). Random additive white Gaussian noise (ranging from 10 to 35 dB) was applied to each image in the training set. The images were symmetrically tiled to create translational invariance and finally normalized to the maximum intensity of the data. To produce the corresponding *k-*space representations for training, each noise-corrupted image was Fourier Transformed with MATLAB’s native 2-D FFT function. The neural network was trained from the noise corrupted *k-*space encodings and target ‘noise-free’ images to learn an optimal feed-forward reconstruction of *k-*space domain into the image domain. RootBox training sets were also generated for matrix size 96 × 96.

### Architecture of neural network

The neural network was trained from the noise corrupted *k-*space encodings and target ‘noise-free’ images to learn an optimal feed-forward reconstruction of *k-*space domain into the image domain. The same network architecture and hyperparameters were used for our experiments as previously described in^10^. The real and the imaginary part of datasets were trained separately. The network was composed of 2 fully connected layers (input layer and 1 hidden layer) of dimension n^2^ × 1 and activated by the hyperbolic tangent function. The 2nd layer was reshaped to n × n for convolutional processing. Two convolutional layers convolved 64 filters of 3 × 3 with stride 1 followed each by a rectifier nonlinearity. The last convolution layer was finally de-convolved into the output layer with 64 filters of 3 × 3 with stride 1. From the real and imaginary trained neural networks, the output layer is either the reconstructed real or imaginary component of the image respectively, which are then combined into the complex image.

For the spike- and noise- corrupted *k*-space database, the neural network architecture was composed of 2 fully connected layers of dimension n^2^ × 1 and activated by the hyperbolic tangent function. The 2nd layer was reshaped to n × n for convolutional processing. Three convolutional layers convolved 128 filters of 3 × 3 with stride 1 followed each by a rectifier nonlinearity. Then the last convolution layer was finally de-convolved into the output layer with 128 filters of 3 × 3 with stride 1. As stated above the real and imaginary components were trained separately and hence the output layer resulted in either the reconstructed real or imaginary component of the image, which are then combined into the complex image.

### Training details

Multiplicative noise at 1% was also applied to the input to learn robust representations from corrupted inputs^10,40^. The RMSProp algorithm was used with following parameters: minibatches size = 100, learning rate = 0.0001, momentum = 0.0, and decay = 0.9. The loss function and the L1 norm penalty applied on the convolutional layer during training were kept the same as described in the previous work^10^. The network was trained for 100 epochs on the Tensorflow deep learning framework using 2 NVIDIA Tesla GV100 GPUs with 16 GB memory capacity each.

### 3D Image reconstruction with AUTOMAP

The in vivo raw datasets of each slice were reconstructed with either AUTOMAP or IFFT. Due to memory limitation of the network architecture of AUTOMAP, we explicitly applied a 1D FFT along the partition direction of the 3D *k-*space, and then applied AUTOMAP to the resultant hybrid-space data to reconstruct images slice-by-slice.

### 2D image reconstruction with AUTOMAP

The raw 2D *k-*space datasets from all samples were stacked and multiplied by a scalar so the range of signal intensities lies within that of the corresponding training models above. The stacked *k-*space were then reconstructed with the corresponding trained model.

### Image analysis

The signal magnitude of each dataset was normalized to unity to enable fair comparison between both reconstruction methods. SNR was then computed by dividing the signal magnitude by the standard deviation of the noise (obtained from a background ROI of the image-space data). For the in vivo brain datasets, error maps were computed using the 22-min scan as the reference image. Image quality metrics were evaluated using RMSE (root mean square error), as well as in-built MATLAB functions—PSNR (Peak SNR), and SSIM (Structure Similarity Index for Measuring image quality).

## Supplementary Information

Supplementary Information
