# Supplementary material for: Boosting the signal-to-noise of low-field MRI with deep learning image reconstruction
Source: Sci Rep. 2021 Apr 15;11:8248. doi: 10.1038/s41598-021-87482-7 (PMC8050246; doi:10.1038/s41598-021-87482-7)
Supplement: Supplementary file 1 — Supplementary Information [file 41598_2021_87482_MOESM1_ESM.docx]

Title

Boosting the signal-to-noise of Low-field MRI with Deep Learning Image Reconstruction

**Authors**

N. Koonjoo,^1, 2^* B. Zhu,^1,2^ G. Cody Bagnall,^3^ D. Bhutto,^1,4^ M. S. Rosen ^1, 2, 5^

^1^Athinoula A. Martinos Center for Biomedical Imaging, Department of Radiology, Massachusetts General Hospital, Charlestown, 02129 MA, USA

^2^Harvard Medical School, Boston, 02115 MA, USA

^3^Department of Biological and Agricultural Engineering, Texas A&M University, College Station, 77843 TX, USA

^4^Department of Biomedical Engineering, Boston University, Boston, 02215 MA, USA

^5^Department of Physics, Harvard University, Cambridge, 02138 MA, USA

*Correspondence to nkoonjoo@mgh.harvard.edu

Supplementary information


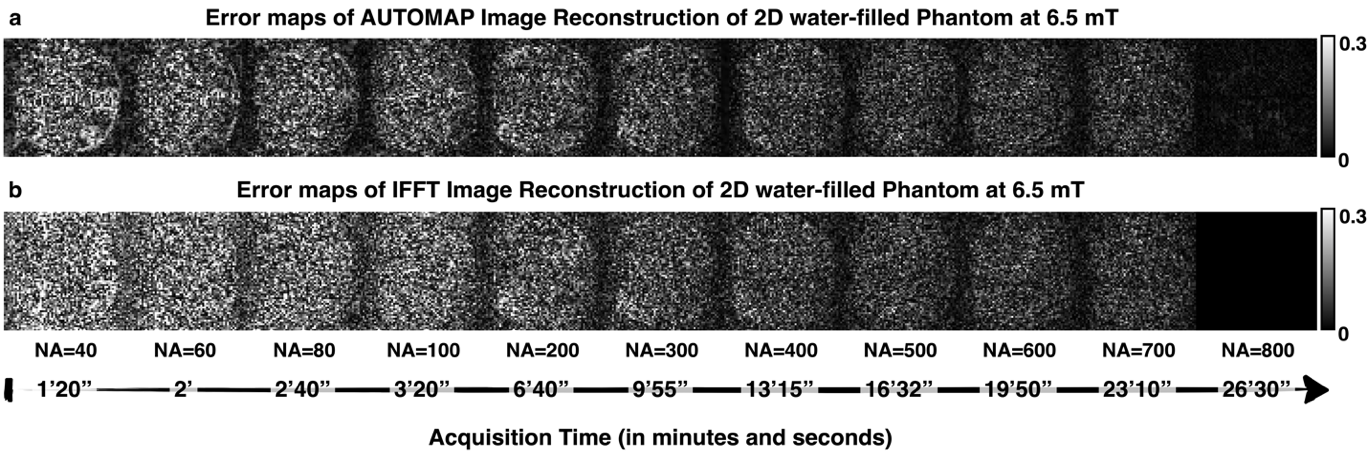


**Fig S1: Error maps of AUTOMAP Image Reconstruction versus error maps of IFFT Reconstruction for 2D phantom images at 6.5 mT.** The NA increases from left to right with their respective scan time below. **a**) Upper panel shows error maps of the AUTOMAP-reconstructed images and **b**) the lower panel shows the error maps computed for same dataset reconstructed with IFFT. The window level of maps (a and b for each NA) is identical. The IFFT reconstructed image with NA=800 was the reference image.
